# Supplementary material for: Pricing strategies for shared manufacturing platform considering cooperative advertising based on differential game
Source: PLoS One. 2024 Jul 10;19(7):e0303928. doi: 10.1371/journal.pone.0303928 (PMC11236110; doi:10.1371/journal.pone.0303928)
Supplement: S1 Data — (DOCX) [file pone.0303928.s001.docx]

Figure 3. Changes in market capacity trajectories over time t

t=0:5:50;

α=5，β=2，η=0.75，δ=0.2，μ_M_=μ_C_=0.5，L_M_=L_C_=1，ρ=0.3，ω=2，Θ=φ=0.4，γ_M_=γ_C_=0.5，K_M_=K_C_=1，W_0_=10，G_0_=15

wwt=(um.^2.*(a-b.*om).*ya.*om)./(2.*lm.*(p+c).*c)+(uc.^2.*ya.*(a-b.*om).^2)./(4.*c.*b.*lc.*(p+c));

y1=wwt+(w0-wwt).*exp(-c.*t);

wwc=(um.^2.*ya.*om.*(a-b.*om))./(2.*c.*lm.*(p+c))+(uc.^2.*ya.*(a-b.*om).^2)./(4.*c.*b.*lc.*(p+c));

y2=wwc+(w0-wwd).*exp(-c.*t);

wwe=um.^2.*((a.^2.*φ+2.*a.*b.*om).*(1-φ).^2-b.^2.*om.^2.*(2-φ)).*ya./(4.*c.*b.*lm.*(1-φ).^2.*(p+c))+uc.^2.*(a.*(1-φ)-b.*om).^2.*ya/(4.*c.*(1-φ).*b.*lc.*(p+c));

y3=wwe+(w0-wwe).*exp(-c.*t);

wwb=(um.^2.*ya.*om.*(a-b.*om))./(2.*c.*(1-thc).*lm.*(p+c))+(uc.^2.*ya.*(a-b.*om).^2)./(4.*c.*b.*(1 -thc).*lc.*(p+c));

y4=wwb+(w0-wwb).*exp(-c.*t);

plot(t,y1,'-r',t,y2,'-*b',t,y3,'-sg');

axis([0,50,0,60]);

Figure 4. The effect of parameters on price

(a)The influence of ɑ and ω parameters on price

x=0:0.5:1;

a=8;b=2;om=2;

y1=(x+b.* om)./(2.*b);

y2=(a+b.*x)./(2.*b);

plot(x,y1,'-r',x,y2,'--b');

xlabel('ɑ/ω');

ylabel('The influence of parameters on price');

legend('The influence of ɑ parameters ','The influence of ω parameters ');

（b）The influence of β parameters on price

x=0:0.5:1;

a=8;b=2;om=2;

y1=(a+x.* om)./(2.*x);

plot(x,y1,'-r');

xlabel('β');

ylabel('The influence of β parameters on price');

（c）The influence of φ parameters on price

x=0:0.5:1;

a=8;b=2;om=2;

y1=(a.*(1-φ)+b.* om)./2./b./(1-φ);

plot(x,y1,'-r');

xlabel('φ');

ylabel('The influence of φ parameters on price');

Figure 5. Profits of Manufacturers and Platforms under the Different Models

(a) Profits under the traditional cooperation model

xn1 = linspace (0 , 2) ;

xn2 = linspace (0 , 1) ;

[ X1 , X2 ] = meshgrid ( xn1 , xn2) ;

y1=10.*(5-2.*X1).*X1.*X2+0.3333.*X1.*X2.^2.*(5-2.*X1).^2.*(1.25-0.25.*X1);

mesh ( X1 , X2 , y1 ,'edgecolor','r') ;

hold on

y2=2.5.*X2.*(5-2.*X1).^2+0.8333.*X2.^2.*(5-2.*X1).^3.*(0.15625+0.1875.*X1);

mesh ( X1 , X2 , y2 ,'edgecolor','g');

view(-150,10);

xlabel('ω');

ylabel('η');

zlabel('Profit');

legend('Manufacturer Profit','Shared Manufacturing Platforms Profit')

(b) Profits under the revenue sharing model

xn1 = linspace (0 , 2) ;

xn2 = linspace (0 , 1) ;

[ X1 , X2 ] = meshgrid ( xn1 , xn2) ;

y1=10.*(5-2.*X1).*X1.*X2+0.125.*X1.^2.*X2.^2.*(5-2.*X1).^2-0.00868.*(5-2.*X1).^4.*X2.^2+0.1042.*(5-2.*X1).^3.*X1.*X2.^2;

mesh ( X1 , X2 , y1 ,'edgecolor','r') ;

hold on

y2=2.5.*X2.*(5-2.*X1).^2+0.013.*X2.^2.*(5-2.*X1).^4+0.125.*(5-2.*X1).^3.*X1.*X2.^2;

mesh ( X1 , X2 , y2 ,'edgecolor','g');

Figure 6. Profit of manufacturers pricing ω under different models

x1=0:0.01:2;

x2=0.75;

y1=10.*(5-2.*x1).*x1.*x2+0.3333.*x1.*x2.^2.*(5-2.*x1).^2.*(1.25-0.25.*x1);

plot(x1,y1,'r');

xlim([0.4,1.4]);

hold on

y2=10.*(5-2.*x1).*x1.*x2+0.125.*x1.^2.*x2.^2.*(5-2.*x1).^2-0.00868.*(5-2.*x1).^4.*x2.^2+0.1042.*(5-2.*x1).^3.*x1.*x2.^2;

plot(x1,y2,'b') ;

hold on

y3=(3.6+7.2.*x1-6.4.*x1.^2).*x2.*[6.944+0.2411.*(3.6+7.2.*x1-6.4.*x1.^2).*x2+0.2893.*(3-2.*x1).^2.*x2];

plot(x1,y3,'g') ;

hold on

y4=10.*(5-2.*x1).*x1.*x2+0.2083.*x1.^2.*x2.^2.*(5-2.*x1).^2-0.00868.*(5-2.*x1).^4.*x2.^2+ 0.1042.*(5-2.*x1).^3.*x1.*x2.^2;

plot(x1,y4,'k') ;

hold off;

d = y1 ./ (y4 + eps);

ix = find(d >.999 & d< 1.001);

x3=x1(ix);

y5=10.*(5-2.*x3).*x3.*x2+0.3333.*x3.*x2.^2.*(5-2.*x3).^2.*(1.25-0.25.*x3);

plot(x1,y1,'r',x1,y4,'k',x3,y5,'rp');

axes('Position',[0.15 0.15 0.3 0.3]);

plot(x1,y1,'r');

hold on;grid on;

plot(x1,y2,'b') ;

plot(x1,y3,'g') ;

plot(x1,y4,'k') ;

axis([0.5,1,20,30]);

axes('Position',[0.15 0.15 0.3 0.3]);

plot(x1,y1,'r');

hold on;grid on;

plot(x1,y2,'b') ;

plot(x1,y3,'g') ;

plot(x1,y4,'k') ;

axis([0.5,1,20,30]);

Figure 7. Profit of shared manufacturing platforms under different models

x1=0:0.01:2;

x2=0.75;

y1=2.5.*x2.*(5-2.*x1).^2+0.8333.*x2.^2.*(5-2.*x1).^3.*(0.15625+0.1875.*x1);

plot(x1,y1,'r');

hold on;

y2=2.5.*x2.*(5-2.*x1).^2+0.013.*x2.^2.*(5-2.*x1).^4+0.125.*(5-2.*x1).^3.*x1.*x2.^2;

plot(x1,y2,'b');

hold on;

y3=0.24.*x2.*(3-2.*x1).^2+0.0217.*x2.^2.*(3-2.*x1).^4+0.0723.*(3.6+7.2.*x1-6.4.*x1.^2).*(3-2.*x1).^2.*x2.^2;%

plot(x1,y3,'g') ;

hold on;

y4=2.5.*(5-2.*x1).^2.*x2+0.013.*x2.^2.*(5-2.*x1).^4-0.3472.*(5-2.*x1).^2.*x1.^2.*x2.^2 +0.1042.*(5-2.*x1).^3.*x1.*x2.^2;%

plot(x1,y4,'k');

xlabel('ω')%('μ_M');

ylabel('Shared Manufacturing Platform Profit');
