# Supplementary material for: Pricing strategies for shared manufacturing platform considering cooperative advertising based on differential game
Source: PLoS One. 2024 Jul 10;19(7):e0303928. doi: 10.1371/journal.pone.0303928 (PMC11236110; doi:10.1371/journal.pone.0303928)
Supplement: Attachment — Submitted filename: Response to Reviewers 1-2 on PONE-D-23-19325.doc [file pone.0303928.s002.doc]

Dear Editor,

Thank you for allowing a major revision of our manuscript (PONE-D-23-19325) for publication in *PLOS ONE.*, with an opportunity to address the reviewers’ comments.

We are uploading (a) our point-by-point response to the comments, (b) an updated manuscript with red highlighting indicating changes, and (c) a clean updated manuscript without highlights.

We thank the referee and the editor very much for the comments and suggestions. We have made considerable effort on revising the paper based on these comments. For future communications, please contact either P. Liu at the addresses listed below.

Yours sincerely,

Peng Liu

Professor Peng Liu

School of Management

Shenyang University of Technology

Shenyang 110870

China

E-mail: liupeng@sut.edu.cn

**Response to the Comments of Reviewer #1 on PONE-D-23-19325**

We thank the referee very much for the comments and suggestions. They are very helpful for us to revise and improve the paper. The paper has been carefully revised according to the referee’s advice. We have made the following changes on the paper accordingly.

**Reviewer#1, Concern # 1:** Overall, I find the topic of this paper to be interesting and a worthy contribution to the literature in this field. I am particularly interested in how the results of this paper may guide corporate and regulatory strategy in the area of shared manufacturing.

**Author response:** We thank the referee very much for the comment.

From Corollary 3 and Figure 2, it can be seen that the revenue-sharing model maintains the highest market capacity in a stable state, followed by the bilateral subsidy model, the cost subsidy model, and finally the traditional cooperation model. Therefore, in order to maintain better advertising results, enterprises in the field of shared manufacturing should give priority to other cooperative advertising models in addition to traditional cooperative advertising; Figure 5 shows the benefits of enterprises in the field of shared manufacturing under different models, from which it can be seen that when the manufacturer's pricing is small, the manufacturer chooses the revenue sharing contract and the profit is higher, and when the manufacturer's price is higher, the bilateral cost subsidy is selected, and the profit is higher.

In general, in the context of the continuous development of shared manufacturing, only by continuously strengthening the source of shared resources and shared media, that is, the cooperative relationship between manufacturers and shared manufacturing platforms, and establishing a sound contract model (such as the bilateral cost-sharing contract and revenue sharing contract in the text), can participants get more benefits with the same advertising investment.

The managerial implications can be found on pages 29-30 in the revised paper.

**Reviewer#1, Concern # 2:** The models proposed are essentially unfounded and unjustified, apart from one reference which remains unexplained. For these results to be of serious interest, the manuscript must go further to explain “why is this model reasonable? Why should a practitioner believe in this model? When does the model break?”

**Author response:** We thank the referee very much for the comment.

As suggested by the referee, we have added the descriptions of research significance in the section “2. Problem descriptions and assumptions”.

This paper considers the convexity of advertising costs, the Nerlove-Arrow model, the linear relationship between price factors and market demand, and completes the model establishment based on the references mentioned in this paper.

[1] Erickson G M. A differential game model of the marketing-operations interface. European Journal of Operational Research,2011,211(2):394-402.

[2] Zhang Jie, Chiang W K, Liang Liang. Strategic pricing with reference effects in a competitive supply chain. Omega,2014,44(2):126-135.

[3] Ouardighi F E. Supply quality management with optimal wholesale price and revenue sharing contracts: a two-stage game approach. International Journal of Production Economics, 2014,156(5):260-268.

[4] Chen G, Zhang X. Research on cooperative advertising strategy in dual-channel supply chain under brand competition. Technology Economics & Management Research, 2019, 5:8-13.

We updated the manuscript with the yellow highlighted changes on pages 8-9 in the revised paper.

**Reviewer#1, Concern # 3:** Experimental results are difficult to interpret. It would help to clearly state the following in each subsection of the numerical results: (a) what is the relationship we should expect from the interaction we are trying to model, (b) how do we construct an experiment to verify that relationship, (c) what is the result, and (d) interpret why the result does or does not support the intuition/expectation. Currently, some subsections state some of these points, but it is inconsistent.

**Author response:** We thank the referee very much for the comment.

As suggested by the reviewers, we added an expectation of numerical results in Section 5, and the results successfully validated the validity of the model established in this paper.

We updated the manuscript with the yellow highlighted changes on page 25 in the revised paper.

**Reviewer#1, Concern # 4:** The abstract and introduction could do a much better job of strongly motivating the topic, orienting the reader to the problem, etc. It is very difficult to parse, as is.

**Author response:** We thank the referee very much for the comment.

As suggested by the reviewer, we have added a description of the importance of cooperative advertising in the introduction.

We updated the manuscript with the yellow highlighted changes on page 1 and page 2 in the revised paper.

**Reviewer#1, Concern # 5:**  The related work discussion is also not doing as good a job as it could: currently, it reads as a list of (seemingly arbitrary) papers with at best a loose organization. This discussion needs to orient the reader first to the general trends of thought in this field, and only then to the (relatively few) specific, closely-related works upon which the present paper is directly based. This structure may be present but it is hidden in lists of references and summaries.

**Author response:** We thank the referee very much for the comment.

As suggested by the referee, we have improved the descriptions of "the current state of literature research" in the introduction.

We updated the manuscript with the yellow highlighted changes on pages 1-3 in the revised paper.

**Reviewer#1, Concern # 6:**  Minor suggestion: use LaTeX for proper equation formatting

**Author response:** We thank the referee very much for the comment. We have improved proper equation formatting.

We updated the manuscript with the yellow highlighted changes on pages 13 -18 in the revised paper.

**Reviewer#1, Concern # 7:**  It seems problematic to assume manufacture production cost is zero. Is this really a good assumption? When does it break?

**Author response:** We thank the referee very much for the comment.

The purpose of this paper is to compare and analyze the decision-making of different models and provide a decision-making basis for shared manufacturing enterprises by establishing models of different cooperation modes and obtaining model equilibrium solutions. Manufacturing costs are not within the range of variables considered in this article, and are equivalent to constants in the calculations and have no effect on the comparison results. This approach to the assumption that the manufacturing cost is zero can also be seen in the following literatures.

[1] Chen G, Zhang X. Research on cooperative advertising strategy in dual-channel supply chain under brand competition. Technology Economics & Management Research, 2019, 5:8-13.

[2] Huo L, Wu J. Research on Competitive advertising strategy of double channel supply chain under influence of goodwill. Computer Engineering and Applications, 2020, 56(07):260-265.

**Reviewer#1, Concern # 8:**  The sharing coefficients in Assumption 4 do not clearly correspond to any model that has been introduced yet. This makes them difficult to interpret.

**Author response:** We thank the referee very much for the comment.

As suggested by the referee, we have improved the descriptions of "Assumption 4 " in the section “2. Problem descriptions and assumptions”. The cost-sharing coefficients of Assumption 4 correspond to the three models of 3.1, 3.2 and 3.4 in this paper, respectively.

We updated the manuscript with the yellow highlighted changes on pages 9-10 in the revised paper.

**Reviewer#1, Concern # 9:**  It is unclear what the authors mean precisely by “two stage Stackelberg game” above Thm. 1. What are the stages, and how do they map onto the continuous time axis? Is this not a standard LQ game for which a feedback Stackelberg equilibrium is sought? What is the information pattern of this game? Similar questions for Thm. 2 and others.

**Author response:** We thank the referee very much for the comment.

As suggested by the referee, we have improved the descriptions of the solution process in the model analysis and solution. The purpose of the model is to determine the optimal level of advertising investment for both the manufacturer and the shared manufacturing platform to maximize their respective profits.

We updated the manuscript with the yellow highlighted changes on page 11 and page 15 in the revised paper.

**Reviewer#1, Concern # 10:**  In Corollary 3, I do not understand the use of the term “saddle point.” If this is describing the graph of a SISO function, it is unclear what function is being referred to. If referring to a game-theoretic equilibrium: are these not general-sum problems (making the term “saddle” inappropriate)? What does “stable” mean in this context?

**Author response:** We thank the referee very much for the comment.

As suggested by the referee, we corrected the word " a stable point " in Corollary 3. The point of the article is that W(t) stabilizes at a fixed value over time.

We updated the manuscript with the yellow highlighted changes on page 22 in the revised paper.

**Response to the Comments of Reviewer #2 on PONE-D-23-19325**

We thank the referee very much for the comments and suggestions. They are very helpful for us to revise and improve the paper. The paper has been carefully revised according to the referee’s advice. We have made the following changes on the paper accordingly.

**Reviewer#2, Concern # 1:** 1. Introduction
a. The introduction section lacks clarity in its presentation, particularly in articulating the significance and novelty of the manuscript.
b. The first important gap in the introduction lies in the limited exploration of the specific dynamics and challenges associated with the intermediary platform among the shared manufacturing platforms. Please improve the introduction section with the addition of relevant business examples.
c. While the study acknowledges its significance, a deeper analysis of the intermediary platform's operational intricacies, unique characteristics, and potential obstacles is needed for a comprehensive understanding. The critical analysis of relevant cases can be added.
d. Another notable gap is the absence of a detailed examination of the effectiveness and impact of cooperative advertising within the context of shared manufacturing platforms, particularly focusing on the intermediary platform. Please add the detailed examination of literature and cases.
e. The comprehensive summary of literature review is missing. Please add the table for the summary of literature review.
f. Please highlight the research gaps and mention research questions, which are not clear in the current form of manuscript.
g. Kindly provide justification for the utilization of cost-sharing, revenue-sharing, and bilateral cost-sharing contracts in the manuscript, supported by relevant business examples and literature.
h. What justifies the application of differential game theory in this paper, and what specific gaps or limitations in existing methodologies or frameworks does the use of differential game theory address within the context of shared manufacturing platforms?

**Author response:** We thank the referee very much for the comment. As suggested by the referee, we have revised the descriptions of Introduction.

a. As suggested by the referee, we have improved the descriptions of " novelty of the manuscript " in the introduction. We updated the manuscript on page 6 in the revised paper.

“In summary, compared with the existing literature on shared manufacturing, the contributions of this paper are: (1) considering the long-term and dynamic characteristics of the impact of advertising investment on product demand, using differential games to study the pricing and advertising strategies under shared manufacturing from the perspective of long-term dynamics; (2) introducing a variety of contracts under the premise of cooperative advertising, and comparing and analyzing the decision-making of shared manufacturing enterprises under different contracts and the impact of different coefficients on the results.”

b. As suggested by the referee, we have improved the descriptions of " the intermediary platform among the shared manufacturing " in the introduction. We updated the manuscript on page 2 in the revised paper.

“This type of intermediary shared manufacturing platform (as shown in Figure 1) does not own its own manufacturing resources and capabilities, such as workers, equipment, and materials. Manufacturing service providers transform idle manufacturing capacity to connect idle manufacturing capacity to the platform to form a virtual "cloud factory". The manufacturing buyer publishes the production order on the platform, and the supply and demand sides finally reach a deal through independent search and negotiation on the platform. The advantages of the intermediary sharing platform are that the platform is more flexible and the user entry threshold is low; The disadvantage is that Internet companies have a lack of understanding of the cross-border manufacturing industry. Typical application cases include "Haizhi Online", which is a leading non-standard parts manufacturing and sharing platform in China, which is mainly responsible for the docking of domestic small and medium-sized parts processing enterprises and global procurement resources. The buyer uploads the parts drawings and order information, calculates the corresponding average market price through the "intelligent price checker", and if the buyer accepts the price, it will match the appropriate factory for it, and finally the factory will make a specific offer. This model helps to shorten the procurement cycle and improve the production efficiency of the factory. In addition, there are Tao factories and Zhibu interconnection.”

c. As suggested by the referee, we have improved the descriptions of " a deeper analysis of the intermediary platforms " in the introduction. We updated the manuscript on page 2 in the revised paper.

d. As suggested by the referee, we have improved the descriptions of " a detailed examination " in the introduction. We updated the manuscript on page 5 in the revised paper.

e. As suggested by the referee, we have added the comprehensive summary of literature review. We updated the manuscript on pages 4-6 in the revised paper.

“From the above literature, it can be seen that the difference between the discussion of pricing in the existing literature is that different state variables are considered, and different platform pricing models are established by changing different charging models or profit subjects. The essence of these models is to analyze the equilibrium solutions of the models under different cost sharing methods or benefit distribution methods, so as to provide a reference for supply chain or shared manufacturing enterprises. Therefore, this paper introduces cost-sharing contracts, revenue-sharing contracts, and bilateral cost-sharing contracts. This paper will deserve further research on its pricing strategy and related decisions.”

“As can be seen from the above literature, the existing literature considers platform pricing decisions under different state variables, but does not share the closer partnerships that manufacturing platforms have emerged with manufacturers in reality. This paper will analyze the pricing decision problem under the advertising cooperation model between the manufacturer and the shared manufacturing platform under different cooperation contracts.”

“The above literature mainly studies the cooperative advertising decisions under the cooperation and competition of different brands in the supply chain, and ignores the introduction of cooperative advertising pricing strategies under different contracts.”

“In summary, compared with the existing literature on shared manufacturing, the contributions of this paper are: (1) considering the long-term and dynamic characteristics of the impact of advertising investment on product demand, using differential games to study the pricing and advertising strategies under shared manufacturing from the perspective of long-term dynamics; (2) introducing a variety of contracts under the premise of cooperative advertising, and comparing and analyzing the decision-making of shared manufacturing enterprises under different contracts and the impact of different coefficients on the results.”

f. As suggested by the referee, we have improved the descriptions of " novelty of the manuscript " in the introduction. We updated the manuscript on page 6 in the revised paper.

“In summary, compared with the existing literature on shared manufacturing, the contributions of this paper are: (1) considering the long-term and dynamic characteristics of the impact of advertising investment on product demand, using differential games to study the pricing and advertising strategies under shared manufacturing from the perspective of long-term dynamics; (2) introducing a variety of contracts under the premise of cooperative advertising, and comparing and analyzing the decision-making of shared manufacturing enterprises under different contracts and the impact of different coefficients on the results.”

g. As suggested by the referee, we have improved the justification in the introduction. We updated the manuscript on page 4 in the revised paper.

“From the above literature, it can be seen that the difference between the discussion of pricing in the existing literature is that different state variables are considered, and different platform pricing models are established by changing different charging models or profit subjects. The essence of these models is to analyze the equilibrium solutions of the models under different cost sharing methods or benefit distribution methods, so as to provide a reference for supply chain or shared manufacturing enterprises. Therefore, this paper introduces cost-sharing contracts, revenue-sharing contracts, and bilateral cost-sharing contracts. This paper will deserve further research on its pricing strategy and related decisions.”

h. Differential games can be solved by multiple decision-makers made in successive periods of time in pursuit of their respective goals, and these decisions influence each other. It has the advantages of dealing with complex systems, making dynamic decisions, and taking into account changes in external factors. On the one hand, considering that this paper involves a shared manufacturing process, there are many parties involved. On the other hand, in order to be realistic, changes in decision-making over time should be taken into account. In conclusion, the advantages of using the differential game method to study cooperative advertising lie in its decision-making process based on a game theory perspective and the application of continuous dynamic game theory.

First, differential games can analyze and solve complex systems involving participants in the shared manufacturing process;

Second, the theoretical basis of differential games allows the consideration of the impact of the different contracts on shared manufacturing, which is helpful to compare decisions in different contract models;

Third, the dynamic nature of the differential game makes the formulation of advertising strategy not only consider the current results but also the long-term significance of advertising decisions.

Therefore, this paper uses differential games to establish a cooperative advertising model under different cooperative advertising models and calculate the decision-making results.

We updated the manuscript with the yellow highlighted changes in the revised paper.

**Reviewer#2, Concern # 2:** 2. Problem descriptions and assumptions
a. What specific gaps in current research exist regarding the selection of cooperation models for advertising investment in shared manufacturing scenarios, considering the leasing of idle resources and the involvement of both the manufacturer and shared manufacturing platform? It is not clear to me, please justify with the relevant business examples.
b. Why is Assumption 1 made, positing that under the cooperative advertising model, the manufacturer and the shared manufacturing platform have distinct advertising goals with a collaborative effect on market expansion and product sales improvement? Are there specific real-world scenarios or settings that validate or necessitate such an assumption in the context of shared manufacturing platforms.
c. Please articulate Assumption 4 clearly, providing a concise explanation supported by transparent cost-sharing equations.

**Author response:** We thank the referee very much for the comment. As suggested by the referee, we have revised the descriptions of Problem descriptions and assumptions.

a. The cost-sharing contract model is that the manufacturer bears a certain coefficient and proportion of the advertising input cost of the shared manufacturing platform; The revenue-sharing contract model is that the shared manufacturing platform will share φ per unit of revenue to the manufacturer according to a certain percentage; In the bilateral cost-sharing contract model, not only the manufacturer share the cost of the shared manufacturing platform, but the shared manufacturing platform also needs to share the cost of the manufacturer. There are some shared platforms such as Predix of GE, Mindsphere of Siemens, ThingWorx of PTC, Tao Factory of Alibaba, and Hai Chuanghui of Haier.

b. As suggested by the referee, we have improved the descriptions of " Assumption 1". We updated the manuscript on page 8 in the revised paper.

“**Assumption 1.** Drawing on the Nerlove-Arrow model to describe the impact of manufacturers and retailers' advertising on market capacity, considering that the advertising investment of manufacturers and retailers will increase market capacity and ultimately affect consumer demand [40,41], the change of market capacity satisfies the following differential equation:

(1)

where δ is the natural decay rate, which represents the impact of advertising on market capacity as it decays over time. And the sensitivity coefficients of market favorability to the advertising effort level of manufacturer and shared manufacturing platform are *μ_M_* and *μ_C_*, respectively. ”

c. As suggested by the referee, we have improved the descriptions of " Assumption 6 (Null Hypothesis 4)". The concise cost-sharing equation is in Assumption 5. We updated the manuscript on page 10 in the revised paper.

“**Assumption 4.** In the revenue-sharing contract model, part of the revenue of the shared manufacturing platform is shared to the manufacturer proportionally, and its sharing coefficient is denoted by φ, where φ∈[0,1] (φ=0 under other models, while φ>0 under the revenue-sharing contract model).

**Assumption 5.** It is assumed that the manufacturer and the shared manufacturing platform make rational decisions based on complete information, and ignore shared manufacturing’s inventory costs and out-of-stock costs. The manufacturer and the shared manufacturing platform have the same discount factor ρ in an infinite time horizon, where ρ>0. Manufacturer pricing is denoted by a constant ω, where 0≤ω≤p.

The profits of the manufacturer and the shared manufacturing platform are as follows:

(4)

**Assumption 6.** In different contract models (the traditional cooperation model, the cost-sharing contract model, and bilateral cost-sharing contract model), the cost-sharing coefficients of the manufacturer and the shared manufacturing platform are ,, respectively.

(1) In the traditional cooperation model, the manufacturer and the shared manufacturing platform do not subsidize the cost of advertising input to each other, namely,,.

(2) In the cost-sharing contract model, the manufacturer shares the advertising input cost for the shared manufacturing platform, namely,.

(3) In the bilateral cost-sharing contract model, the manufacturer and the shared manufacturing platform share the cost of advertising input with each other, namely, .”

We updated the manuscript with the yellow highlighted changes in the revised paper.

**Reviewer#2, Concern # 3:** 3. Model analysis and solution
a. Clarify the process of deriving all equilibrium results and ensure that detailed proofs for all theorems are included. Currently, only the proof for Theorem 1 is found in the main body of the manuscript. Consider presenting important results in the main body and relocating all proofs to the appendix or supplementary section.
b. The detailed proofs for all corollaries are absent. It is recommended to present proofs in the appendix or supplementary section, with comprehensive explanations included in the main body of the manuscript, following the convention observed in high-quality journals.
c. Given Corollary 1, which asserts that as market development capabilities increase, advertising efforts by the manufacturer and shared manufacturing platform also increase, and that higher manufacturer pricing leads to an initially increasing and then decreasing trend in advertising efforts and resulting profits, what real-world cases or empirical evidence can be cited to justify these observed trends?
d. How do these dynamics align with practical scenarios, and can existing business cases provide insights into the implications of market, and profits in shared manufacturing platforms? Please justify all theorems and corollaries.

**Author response:** We thank the referee very much for the comment. As suggested by the referee, we have revised the descriptions of Model analysis and solution.

a. As suggested by the referee, we have improved the descriptions of " proof for Theorems ". We updated the manuscript on pages 11-20 in the revised paper.

b. As suggested by the referee, we have improved the detailed proofs for all corollaries. We updated the manuscript on pages 11-20 in the revised paper.

c. As suggested by the referee, we have improved the descriptions of " Corollary 1”. We updated the manuscript on page 13 in the revised paper.

Corollary 1.

(1)“The sensitivity coefficients of market favorability to the advertising effort level, μ_M_ and μ_C_， is directly proportional to the advertising efforts E_M_ and E_C_”: Ref. [1] can demonstrate.

(2)“As manufacturer pricing, constant ω, increases, the manufacturer's profit under the traditional cooperation model increases.”: Ref. [2] can demonstrate.

Corollary 2.

“The market capacity of the cooperation model is monotonic”: Ref. [3] can demonstrate.

“the price has no effect on the optimal trajectory of market capacity and its stable value.”: Ref. [4] can demonstrate.

[1] Huo L, Wu J. Research on Competitive advertising strategy of double channel supply chain under influence of goodwill. Computer Engineering and Applications, 2020, 56(07):260-265.

[2] Yao F, Liu S, Sun J, Teng C. Advertising and pricing decision models for closed-loop supply chain based on third-party collecting. Computer Integrated Manufacturing Systems, 2019, 25(09):2395-2404.

[3] Ye X, Zhou Y. Dynamic pricing and joint emission reduction strategy in a dual-channel supply chain considering goodwill. Chinese Journal of Management Science, 2021, 29(02):117-128.

[4] You D, Zhu G. Differential game analysis of ecological R&D, cooperative promotion and pricing in the low-carbon supply chain. Control and Decision, 2016, 31(06):1047-1056.

d. We thank the referee very much for the comment.

Lu et al. [1] used the differential game model to study two cooperation models (cost-sharing and benefit-sharing contracts), and analyzed the different coordination capabilities of the two contracts. On the basis of the cost-sharing model, Kunter [2] and Zhang et al. [3] conducted an in-depth study on the participation rate of two bilateral subsidy strategies for cooperative advertising, and found that an appropriate bilateral subsidy strategy can coordinate the supply chain and improve the efficiency of the supply chain.

The insights on advertising investment and the establishment of models and profit analysis in these literatures can justify the inferences of this paper

[1] Lu X，Li Y，Wang J，Yu S. Differential game analysis of technological innovation and dynamic pricing in the cloud service supply chain. Operations Research and Management Science, 2020, 29(06):49-57.

[2] Kunter, M. (2012). Coordination via cost and revenue sharing in manufacturer-retailer channels[J]. European Journal of Operational Research,216(2),477-486.

[3] Zhang, J., Xie, J., & Chen, B. (2013). Cooperative advertising with bilateral participation[J]. Decision Sciences, 44(1), 193-203.

We updated the manuscript with the yellow highlighted changes in the revised paper.

**Reviewer#2, Concern # 4:** 4. Comparative analysis
a. Please explain the Comparative analysis in more detail citing relevant business examples.
b. In the Table 4, increasing, decreasing, and no change representation is not clear, for example, p^(T*) is increasing with increase in α or decrease in α. Please make the table more comprehensive by adding nature of change of α, β, ω, and φ.

**Author response:** We thank the referee very much for the comment.

a. The comparative analysis in the section 4 was conducted theoretically, and numerical example simulation analysis was conducted in the section 5.

b. As suggested by the referee, we have improved the descriptions of “Table 4”. We updated the manuscript on pages 22-25 in the revised paper. As suggested by the referee, we have improved the detailed proofs for all corollaries. We updated the manuscript on pages 21-24 in the revised paper.

We updated the manuscript with the yellow highlighted changes in the revised paper.

**Reviewer#2, Concern # 5:** 5. Numerical Analysis
a. Please add more examples for setting the value of relevant parameters. Are all parameters in line with first order and second order conditions?
b. Please enhance the clarity and quality of the figures, particularly the line representing bilateral cost-sharing, which is currently unclear (in Figure 2). Consider adding legends to distinguish between multiple lines for improved interpretation.

**Author response:** We thank the referee very much for the comment. As suggested by the referee, we have revised the descriptions of Numerical Analysis.

a. Based on the literature [25, 33], we set the relevant parameter. All parameters are in line with first order and second order conditions.

b. As suggested by the referee, we have enhanced the clarity and quality of the figures. We updated the manuscript on page 25 in the revised paper.

We updated the manuscript with the yellow highlighted changes in the revised paper.

**Reviewer#2, Concern # 6:** 6. Conclusion
a. The conclusion section requires substantial improvement, particularly in terms of the quality of presentation, with a focus on clearly articulating the significant contributions of the study on the pricing strategies for shared manufacturing platform.
b. Consider incorporating two subsections in the manuscript: one to emphasize theoretical contributions and another to address practical/managerial implications. The current version lacks these essential components.

**Author response:** We thank the referee very much for the comment. As suggested by the referee, we have revised the descriptions of Conclusion.

a. As suggested by the referee, we have revised the descriptions of Conclusion. We updated the manuscript on page 29 in the revised paper.

“Based on the theory of cooperative advertising, this paper introduces the sensitivity coefficient of the market to the advertising efforts of manufacturers and shared manufacturing platforms, and studies the optimal pricing strategy of shared manufacturing platforms. By establishing the traditional cooperation model, the cost-sharing contract model, the revenue-sharing contract model and the bilateral cost-sharing contract model, we discuss the influence of manufacturers' pricing, market sensitivity coefficient to advertising efforts and time on the equilibrium profit and optimal pricing decision of the shared manufacturing platform. And specific conclusions are given.”

b. As suggested by the referee, we have revised the descriptions of Conclusion. We updated the manuscript on page 30 in the revised paper.

“In conclusion, this paper analyzes the optimal pricing strategy of the shared manufacturing platform under different contract modes based on the cooperative advertising theory, and provides a theoretical reference for the shared manufacturing platform to formulate a reasonable pricing strategy. The impact of platform pricing and the market demand of manufacturers is also considered when establishing the pricing model, which is in line with the actual situation of the platform operation, which makes the pricing model in this paper more realistic.”

We updated the manuscript with the yellow highlighted changes in the revised paper.
