# Supplementary material for: Pricing strategies for shared manufacturing platform considering cooperative advertising based on differential game
Source: PLoS One. 2024 Jul 10;19(7):e0303928. doi: 10.1371/journal.pone.0303928 (PMC11236110; doi:10.1371/journal.pone.0303928)
Supplement: Attachment — Submitted filename: Response to Reviewers 1-3 on PONE-D-23-19325R1.doc [file pone.0303928.s003.doc]

Dear Editor,

Thank you for allowing a major revision of our manuscript (PONE-D-23-19325R1) for publication in *PLOS ONE.*, with an opportunity to address the reviewers’ comments.

We are uploading (a) our point-by-point response to the comments, (b) an updated manuscript with red highlighting indicating changes, and (c) a clean updated manuscript without highlights.

We thank the referee and the editor very much for the comments and suggestions. We have made considerable effort on revising the paper based on these comments. For future communications, please contact either P. Liu at the addresses listed below.

Yours sincerely,

Peng Liu

Professor Peng Liu

School of Management

Shenyang University of Technology

Shenyang 110870

China

E-mail: liupeng@sut.edu.cn

**Response to the Comments of Reviewer #1 on PONE-D-23-19325R1**

We thank the referee very much for the comments and suggestions. They are very helpful for us to revise and improve the paper. The paper has been carefully revised according to the referee’s advice. We have made the following changes on the paper accordingly.

**Reviewer#1, Concern # 1:** Thank you very much for addressing my comments, and particularly for the work you have done to clarify modeling assumptions.

**Author response:** We thank the referee very much for the comment.

**Response to the Comments of Reviewer #2 on PONE-D-23-19325R1**

We thank the referee very much for the comments and suggestions. They are very helpful for us to revise and improve the paper. The paper has been carefully revised according to the referee’s advice. We have made the following changes on the paper accordingly.

**Reviewer#2, Concern # 1:** I am perfectly satisfied with the quality of revision, and accept the manuscript.

**Author response:** We thank the referee very much for the comment.

**Response to the Comments of Reviewer #3 on PONE-D-23-19325R1**

We thank the referee very much for the comments and suggestions. They are very helpful for us to revise and improve the paper. The paper has been carefully revised according to the referee’s advice. We have made the following changes on the paper accordingly.

**Reviewer#3, Concern # 1:** Abstract does not clearly reflect the innovation and importance of the research, it can be summarized better.

**Author response:** We thank the referee very much for the comment.

As suggested by the reviewer, we have added a description of the innovation and importance of the research in the abstract.

We updated the manuscript with the yellow highlighted changes on page 1 in the revised paper.

**Reviewer#3, Concern # 2:** The introduction should be summarized better and stimulate the topic better.

**Author response:** We thank the referee very much for the comment.

As suggested by the reviewer, we have added a description in the introduction.

We updated the manuscript with the yellow highlighted changes on page 6-7 in the revised paper.

**Reviewer#3, Concern # 3:** Figure 1 includes the retailer, why is the price p in equation 2 not the retailer pricing, and it's about Shared manufacturing platform pricing?

**Author response:** We thank the referee very much for the comment.

Figure 1 illustrates the entire operating model of an intermediary shared manufacturing platform, including manufacturers, shared manufacturing platforms, and retailers. In this paper, a secondary model consisting of a shared manufacturing platform is established. We use this model to analyze their interests and cooperation. And the retailers' demand is influenced by the price of a shared manufacturing platform. So the price p in equation 2 is not the retailer pricing, and it's about shared manufacturing platform pricing.

**Reviewer#3, Concern # 4:** Why do the hypothesis 2 appear Dw and DG?

**Author response:** We thank the referee very much for the comment. We have changed“$D_{W},D_{G}\geq0$”to*“*$D\left(W,P\right)\geq0$” in the revised paper.

We updated the manuscript with the yellow highlighted changes on page 9 in the revised paper.

**Reviewer#3, Concern # 5:**  It seems to be something wrong with equation 4.

**Author response:** We thank the referee very much for the comment.

In the revised paper, we put Equation 4 in Assumption 6. The coefficients,$\theta_{M}$and $\theta_{C}$ , are explained in Assumption 5. Compared to the original paper, we adjusted the order of the assumptions 5 and 6 in the revised paper. And there is no problem with Equation 4 after the adjustment.

We updated the manuscript with the yellow highlighted changes on pages 9-10 in the revised paper.

**Reviewer#3, Concern # 6:**  In Corollary 5, the authors mentioned that “With the increase of the manufacturer's price ω, the profit of the manufacturer increases first and then decreases”, Can you figure out the range of ω?

**Author response:** We thank the referee very much for the comment.

As suggested by the reviewer, we have added the range of ω in Corollary 5.

We updated the manuscript with the yellow highlighted changes on page 24 in the revised paper.

**Reviewer#3, Concern # 7:** The ordinates of Figure 3(a), Figure 3(b), and Figure 3(c) are not clear.

**Author response:** We thank the referee very much for the comment.

We updated the manuscript with the yellow highlighted changes on pages 26-27 in the revised paper.

**Reviewer#3, Concern # 8:**  More management implications can be added to the conclusion.

**Author response:** We thank the referee very much for the comment.

We added management implications in the conclusion.

We updated the manuscript with the yellow highlighted changes on pages 30-31 in the revised paper.
